# Entrepreneurship beyond the lab: commercializing your creative outputs

**DOI:** 10.1186/s12919-026-00383-3

**Published:** 2026-06-16

**Authors:** Michelle M. Martínez-Montemayor, Surya Raghu, Annelyn Torres-Reverón

**Affiliations:** 1 Dynamiko Pharmaceutics, LLC, 60 W Churchill 501E, San Juan, 00926 Puerto Rico; 2 Department of Biochemistry, Universidad Central del Caribe, Bayamón, 00960 Puerto Rico; 3 Advanced Fluidics, LLC, Columbia, MD 21045 United States; 4 Sur180 Therapeutics, Inc., McAllen, TX 78504 United States; 5 Ponce Health Sciences University-Ponce Research Institute, Ponce, 00716 Puerto Rico

**Keywords:** Entrepreneurship, Intellectual property, Innovation, Commercialization, Biotechnology, Patent, Accelerator, Technology transfer, Small business, Licensing

## Abstract

Scientists invest years and decades in research discoveries that may lead to important life-saving technologies that benefit humanity. However, many of these researchers may not be familiar with determining whether their ideas can be turned into a viable product and made sellable. Educational programs such as the National Science Foundation I-Corps Program and I-RED from the National Institute of General Medical Sciences at the NIH support this shift toward faculty gaining greater entrepreneurship knowledge. These programs provide additional preparation for faculty, particularly in specialized business information and entrepreneurial skills. Despite the importance of commercialization training, few post-doctoral and faculty members in life sciences receive formal education in intellectual property, entrepreneurship, and research commercialization. Accordingly, this article aims to provide a guide to help start or facilitate the entrepreneurial journey to support the commercialization of their creative outputs. The article provides a timeline of events from idea discovery to technology commercialization, translating academic skills into entrepreneurial careers, understanding the entrepreneurial ecosystem, and discussing the pros and cons of entrepreneurship for scientists. The article emphasizes the need for academic researchers to gain knowledge in research commercialization and entrepreneurship to impact and benefit humanity beyond the laboratory.

**Supplementary Information:**

The online version contains supplementary material available at 10.1186/s12919-026-00383-3.

## Background

Scientists dedicate decades of effort towards conducting impactful scientific research that may provide life-saving discoveries at the university. In many cases, these discoveries make priceless contributions to humanity; thus, many researchers may decide to take the next steps toward commercializing them [1]. Scientists who decide to take the entrepreneurial road at the university may need to develop business skills to commercialize creative outputs generated at their institutions.

Although shifting from academia to the entrepreneurial world may seem overwhelming, scientists possess many qualities and skills that are transferable to this new endeavor. Some challenges that scientists may face during this time include not being familiarized with the timeline of events that lead from idea to product, or the steps needed to secure protection of their inventions and discoveries, steps to form a company, funding sources that companies may seek, and the skill set required to shift from academia to the entrepreneurial world. Very few postdoctoral trainees and faculty in the life sciences receive training in intellectual property (IP), entrepreneurship, and research commercialization, as these topics are rarely taught as formal courses for scientists-in-training at most universities. However, it is increasingly important and relevant for university researchers and faculty to receive training in commercialization and entrepreneurship to diversify the research portfolio. Furthermore, research topics could be selected from pressing societal needs, as indicated by Pasteur’s quadrants [2]. For example, the coronavirus pandemic crisis is a case in point and served as an excellent example of the rapid development of diagnostic testing methods for quick screening of diseases in suspect populations.

Many developed IP forms that are created in academia can be commercialized, including research outputs, unique and novel laboratory procedures, evaluation and survey methods, data analysis methods, and representations or graphics [3]. For this reason, there is an increased need for academic researchers to receive knowledge of essential research commercialization and entrepreneurship processes. These scholars can likely commercialize some of their creative outputs at the bench to have an impact beyond the laboratory. Accordingly, in this article, we provide information and a series of steps to guide scientists on their entrepreneurial journey toward the commercialization of their creative products.

The goals of this manuscript are:

1. Provide a guide with a generalized set of steps towards the entrepreneurial journey (timeline provided).
2. Identify the skills that directly translate from the lab to the business venture.
3. Provide a brief overview of the entrepreneurial ecosystem.
4. Introduce any career-level scientists in academia (late postdoc, early faculty, or experienced faculty in entrepreneurship) to the skills and ecosystems (issues/challenges) associated with establishing your own biotech company and/or commercializing your scholarly discoveries in the United States.

## Timeline to consider when shifting into the entreprenurial journey

**Fig. 1:**
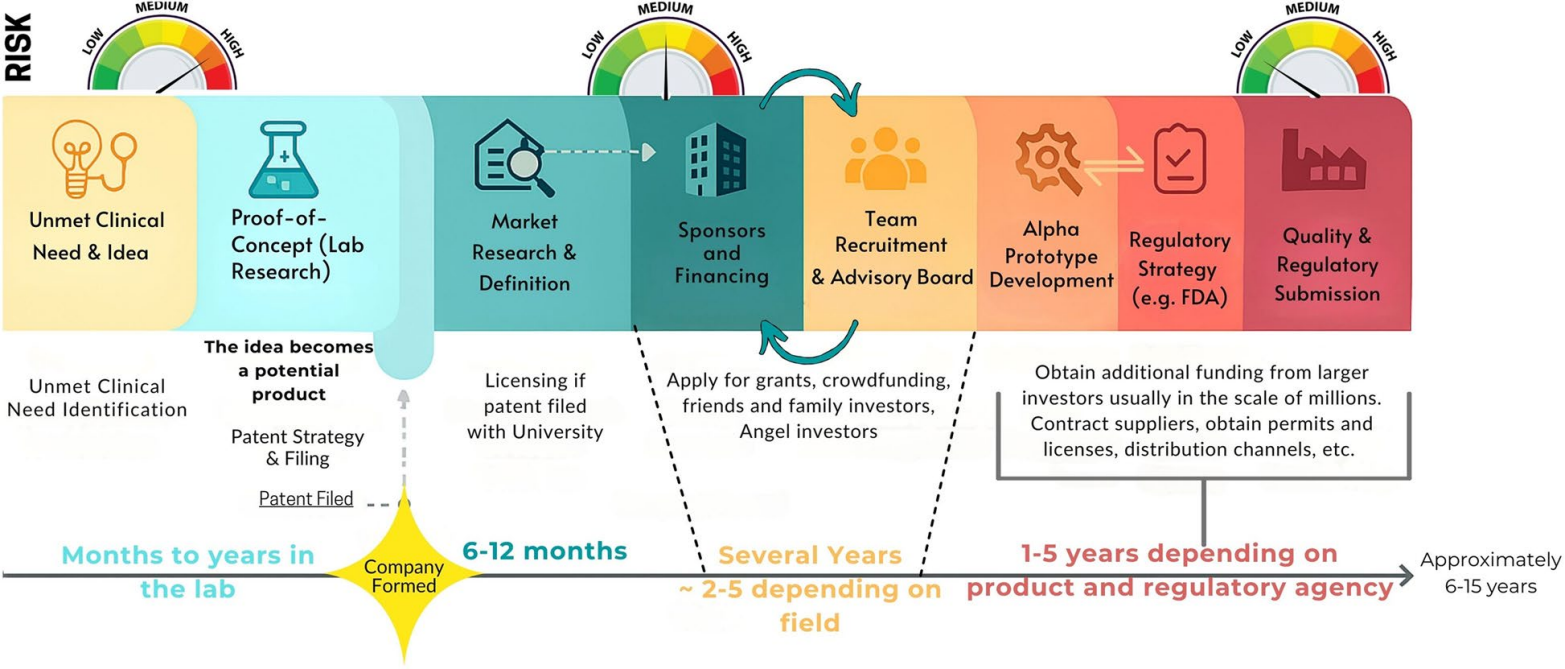
Timetable when considering a shift towards the entrepreneurial journey. This figure was created as an example based on the development of a medical device

Transitioning from academia to the business world begins with identifying a solution to an unmet clinical need (Fig. 1). The discovery made in the laboratory must provide a concrete idea that addresses a real-world problem or need, and this idea should have the potential to become a viable product in the market. For months to years, this idea has been researched in the laboratory, and after demonstrating proof of concept in the laboratory, the results of this discovery should be disclosed to the university's technology transfer office (TTO) to identify the appropriate IP protection strategy. This is usually done through an “invention disclosure form”. This begins the process of protecting your discovery to commence the commercialization journey. The TTO generally evaluates the invention disclosure to determine the best way to protect the IP and assess the innovation's commercialization potential. Also, note that any IP protection filed through the university is owned by the university. Upon receiving feedback from the TTO and, occasionally, on the scientist’s own entrepreneurial experience, contacts, research, and drive to commercialize, the scientist may consider forming a company (e.g., an LLC or C-Corp) for this purpose and plan a licensing arrangement with the university for the IP. The initial stage of the commercialization process poses the greatest risk due to numerous factors and uncertainties: financial risk (balancing commitment from current job and new role), labor wise (employment, personal dynamics), funding risks, technological risk (similar solutions for the problem from other companies), data innovation cycles (many things are happening around the world at a faster rate). Statistics show that a significant percentage of startups fail within the first few years. This high-risk phase also includes de-risking the technology to the point that it is ready for the market that will be pursued. At some universities, TTOs help early-stage entrepreneurs navigate this high-risk phase by providing them with access to Technology Incubators owned by or partnering with the university. In such cases, significant assistance is also provided for market analysis, funding sources, legal services, and networking opportunities.

The next phase (often called the “customer discovery phase”) involves defining the market the company will enter. This is essential to define because it represents the technology's potential buyers. These customers share the need for the technology being created and have the capacity to buy your product. By this time, the company may license the technology to obtain the rights and proceed with commercialization. Importantly, once the technology's licensing is negotiated, the company may be responsible for patent costs. Therefore, the company should have recurring funding and be sustainable to carry on the commercialization process. The company may seek funding from sources such as small business grants, crowdfunding, and various types of investors (friends and family, angel investors, venture capital, etc.). During this phase, the company will grow in size as it recruits additional team members, for example, members of the advisory board. This is a medium-risk phase that may last 5–6 years, depending on the field type that the technology serves.

Finally, once the technology has passed the proof-of-concept stage and is ready for prototype development, the company may begin forming partnerships or seeking additional funding. This phase involves defining the Food and Drug Administration (FDA) regulatory strategy for the technology approval. Companies will contract suppliers, obtain the necessary permits and licenses, and establish distribution channels for product sales. This phase involves a lower risk and may take 1–5 years to complete, depending on the product and the regulatory agency.

## Translatable skills from the lab to the business venture

Scientists with thriving laboratories often possess characteristics highly transferable to tasks needed by principal investigators pursuing the business path. Mentoring and supervision, project management, scientific and lay-language communication, networking, presentation skills, fundraising, budget management, and securing resources to conduct research are among the knowledge, skills, and abilities needed to start, manage, and run a business successfully.

*Foundational Knowledge*: Scientists who are part of developing businesses have a defined idea (technology) that will solve a problem (unmet clinical need). Therefore, the fundamental information that provides the scientific basis for the technology that will address the unmet clinical need is essential, as entrepreneurship will be at the center of business development. The scientific knowledge developed in the laboratory will nourish your startup, and over time, additional data will sustain your company’s opportunity to evolve into a sustainable business. In addition, a willingness to learn new things outside of your scientific expertise – such as good manufacturing practices (GMP), manufacturing, supply chains, software, etc. is vital for an entrepreneur. Accordingly, the startup should have a solid foundation backed by publications, expertise, and grants, which helps them to gain what is known in the entrepreneurial world as traction [4]. Traction refers to the startup's progress and momentum in gaining customer interest, user engagement, market demand, and revenue generation. Examples of traction include increases in sales or revenue over a specific period, which can be illustrated with a line chart showing the trend over time. Real-world examples include Genentech, a rare success story in the biotechnology industry, founded in 1976. The founders, Herbert Boyer and Robert Swanson, had limited financial resources, so they turned to Tom Perkins, the co-founder of Kleiner Perkins, for venture capital. Genentech was developed through an effective union between scientific and venture investment mindsets, and in 1980, an initial public offering (IPO) valued Genentech at $300 million [5]. Another example is the story of Moderna, which was founded by Robert Langer. Dr. Langer is a chemical engineer at the Massachusetts Institute of Technology (MIT) in Cambridge, and one of the researchers behind one of the vaccines developed for COVID-19. According to Martin Bliemel, acting associate dean of research at the Faculty of Transdisciplinary Innovation at the University of Technology, Sydney, Australia, the elements for entrepreneurship that increase traction, as seen in Langer, include think big, patent broadly, publish strategically, and build the founding team with care [6]. Therefore, it is essential to highlight that potential investors are interested in the team, and this requires a foundation built on solid science and productivity.

**Fig. 2:**
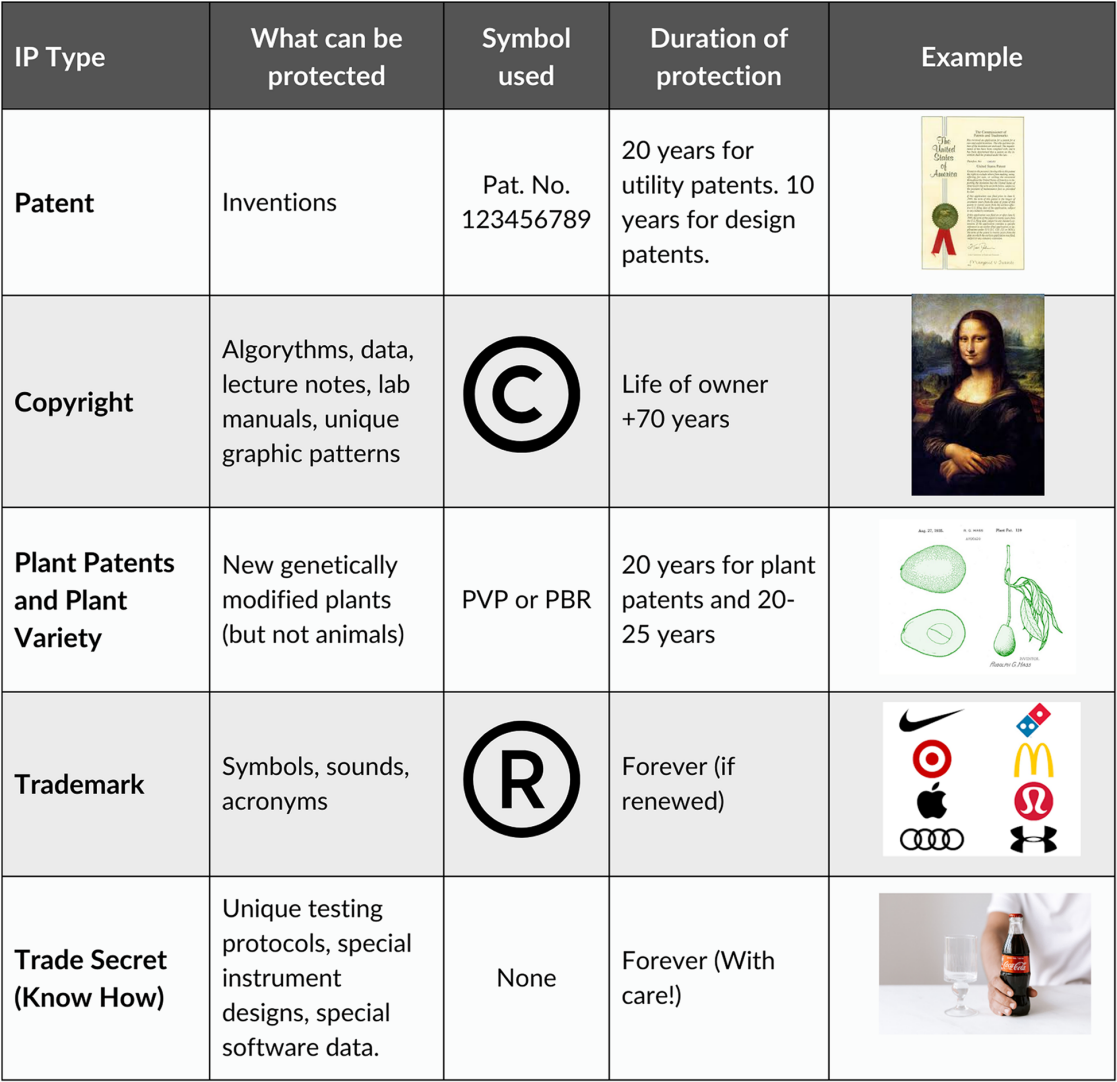
Basic forms of intellectual property (IP)

*Intellectual property (IP)* (Fig. 2): It is imperative to protect your technology and understand the implications of protection, term, type, and where it is protected. Innovative solutions resulting from your research can be protected as IP if they have commercial potential. This commercialization can happen through licensing by your TTOs or through a start-up emerging from your Lab member. Figure 2 describes basic common forms of IP related to university research that you may find helpful.

Patenting is the most common route sought for protecting inventions at the university [7]. Usually, the inventor completes an invention disclosure form (some universities call it “innovation disclosure form”), which the TTO then evaluates for its patentability. The TTO performs a thorough patent and literature search to determine if the information can be pursued for patent filing. If so, the TTO files a provisional application with the United States Patent and Trademark Office (USPTO) describing the invention. The date of filing this application is the “priority” date, after which the university has one year to file the full patent application. During this period, all the information required for filing the patent is collected by the TTO in cooperation with the inventor(s), and subsequently, a full application for a patent is filed by the TTO within the year.

It is strongly advised that a “prior art” search be conducted before the full (non-provisional) application is filed. Prior art can exist in either patents, published patent applications, scientific and technical literature, conference or video presentations. The USPTO website is a very good place to search for prior art in patents and pending patent applications. Proper search terms – either by industry, branch of engineering or science, application, or authors (if known) would greatly help the prior art search process. In addition, artificial intelligence (AI) tools (e.g., Google Patent Search and private companies) are increasingly common search tools.

One important note here is that the order of the inventors’ names in the patent application does not imply seniority, as it does in publications where the first author is the principal author. All inventors listed on the patent will have equal rights to the patent. It is also illegal to list members who did not contribute to the invention described in the patent, and the patent can be annulled in the event of a lawsuit over the same. If you are employed by the university, generally the university maintains patent ownership (by policy) unless it is explicitly negotiated that the faculty member is given the right to own the patent.

### Types of patents

The most common type of patent filed is the utility patent [8]. This covers the entire invention, for new processes, machines, articles, compositions of matter, or improvements thereof, a method based on the application of science and technology. This type of patent should contain novelty and an innovative step that is considered “non-obvious” for someone skilled in the art. Hence, considerable rigor is needed in both the innovative work and the description of the novelty and innovative step in the patent application. Utility patents have a duration of 20 years from the date the application was filed, including the provisional application.

Another type of patent in the US Patent system is the Design Patent. These patents cover a particular shape, geometry, or layout used in a product and do not cover the function of each shape applied for in the patent. For example, electronic chip layouts, external shapes, textures, colors, and combinations of color and appearance of an instrument or product can be protected by design patents. In universities, these types of patents are less likely to be used since the final product is usually designed by a business after full customer consideration.

At universities that research plants and cells, inventors can obtain patents for inventions or discoveries involving plants that can be reproduced asexually (35 U.S. Code § 161–164). This includes cultivated sports, mutants, hybrids, and newly found seedlings. Asexually propagated plants are plants that are reproduced by means other than from seeds, such as cutting, layering, grafting, division, or cloning. The Plant Variety Protection Act (7 U.S. Code Chapter 57) is a law that provides intellectual property rights protection to plant breeders who create new varieties of seed-propagated and tuber-propagated plants and asexually reproduced plants. The Plant Variety Protection Office administers the Plant Variety Protection Act.

Algorithms, special testing methodologies (e.g., for clinical trials), data analytics, unique graphic patterns [University of Florida Tie patterns, Institute of Physics (IOP) tie patterns], and lab procedures, lecture notes developed at the university can be protected by registered copyright. Occasionally, universities have filed for trademarks [e.g., Gatorade (U. of Florida)], Taxol – Research Triangle Institute International and marketed them to companies. The hardest type of IP protection in universities is the “know-how” of “trade secrets”. Universities are meant to be a place of open research and academic freedom. Students work in research labs and gain significant knowledge and skills in the techniques used in such environments, and, rightly, they have the right to learn as much as they can during their tenure. Only when they apply their skills outside the university (referred to as “knowledge transfer”) is the university's purpose served.

### Pitfalls in patent filing

1. Premature provisional application: This results in limiting the number of claims, as new aspects of the invention cannot be added to a submitted provisional application during the non-provisional phase of the application. The new aspect of the invention will not be given the old priority date, and this can complicate the patent filing.
2. Early disclosure through conferences and journal publications may result in the patent not being issued since these instances both constitute public disclosure. A good practice would be to send the abstracts, manuscripts, posters, or oral presentations to the TTO before submitting publications or presentations to obtain advice on IP protection.
3. Even if part of the invention is disclosed in public, it will result in a very narrow set of claims, rendering the patent almost worthless from the perspective of commercialization.
4. Patenting, whether by a university or individuals, is an expensive process, particularly if one needs international coverage – not only during patent drafting and filing, but also all assistance provided by the patent attorney and the maintenance cost of the patents. Hence, unless there is commercial interest, both universities and start-ups wait it out until they have customers ready to license or buy the product.
5. If the IP is licensed, the terms of licensing are very important so as not to lose the right to work in that area and file for further patents without permission from the licensee.

Hence, the IP behind your technology, the market you will embark on, your competition, and your technology’s value proposition are all part of the knowledge you can transfer from the bench to the business. A value proposition conveys the scientific metrics and merits of your innovation in a language the customer understands and appreciates, motivating them to buy or use the product or service.

### Skills

1. Adaptability – Researchers think fast and adapt quickly to different environments. Thus, adaptability is instrumental when deciding to shift from the laboratory to a business setting, as businesses require flexibility. Striking a balance between scientific perfection and what is reasonably sufficient in a product (good enough) is a significant shift in mindset or adaptability that researchers need to become entrepreneurs. In addition, appreciating market forces, competition, and technological advancements outside your world that could adversely impact your product and market – all bundled into a “risk factor” – is essential for an entrepreneurial journey.
2. Soft skills (sociability and networking) – The business world requires active networking and social skills. Because investors primarily invest in the team, researchers who embark on entrepreneurship must be ready to present themselves and their technology using simple terminology in highly social settings.
3. Grant writing – This skill is necessary to secure funds for the business, especially non-dilutive funds from Small Business Innovation Research (SBIR) / Small Business Technology Transfer Research (STTR) grants. Whether it is in the initial stages of your startup or because you have a new idea that needs financing, non-dilutive funding allows startups to retain full ownership of their company.
4. Project management – Organization as a principal investigator and entrepreneur is essential to delivering a product, pitching, or securing funding. Therefore, one must be constantly aware of deadlines and task completion, lead experiments, and ensure that projects are completed according to the plan established in both the short- and long-term.

### Abilities

1. Communication – Clear and constant communication with your team, even if it's a two-person team. Continuous and transparent communication is essential for a successful business venture.
2. Leadership is necessary for the development of the enterprise, the achievement of the proposed research projects, and the completion of the product.
3. Time management skills are key, especially for entrepreneurs who run laboratories at universities and have created a startup, as well as for businesspeople who work full-time in business. All tasks at hand need to be carried out promptly. Spreadsheets, color-coded calendars, reminders, organizational programs, and alerts are great tools for managing and completing tasks.
4. Creativity – generating high-risk, innovative, and sustainable ideas that can be executed in the startup adds value to the business.

## Brief overview of the entrepreneurial ecosystem

### Understanding your local business ecosystem and networking with the members of the ecosystem

**Fig. 3:**
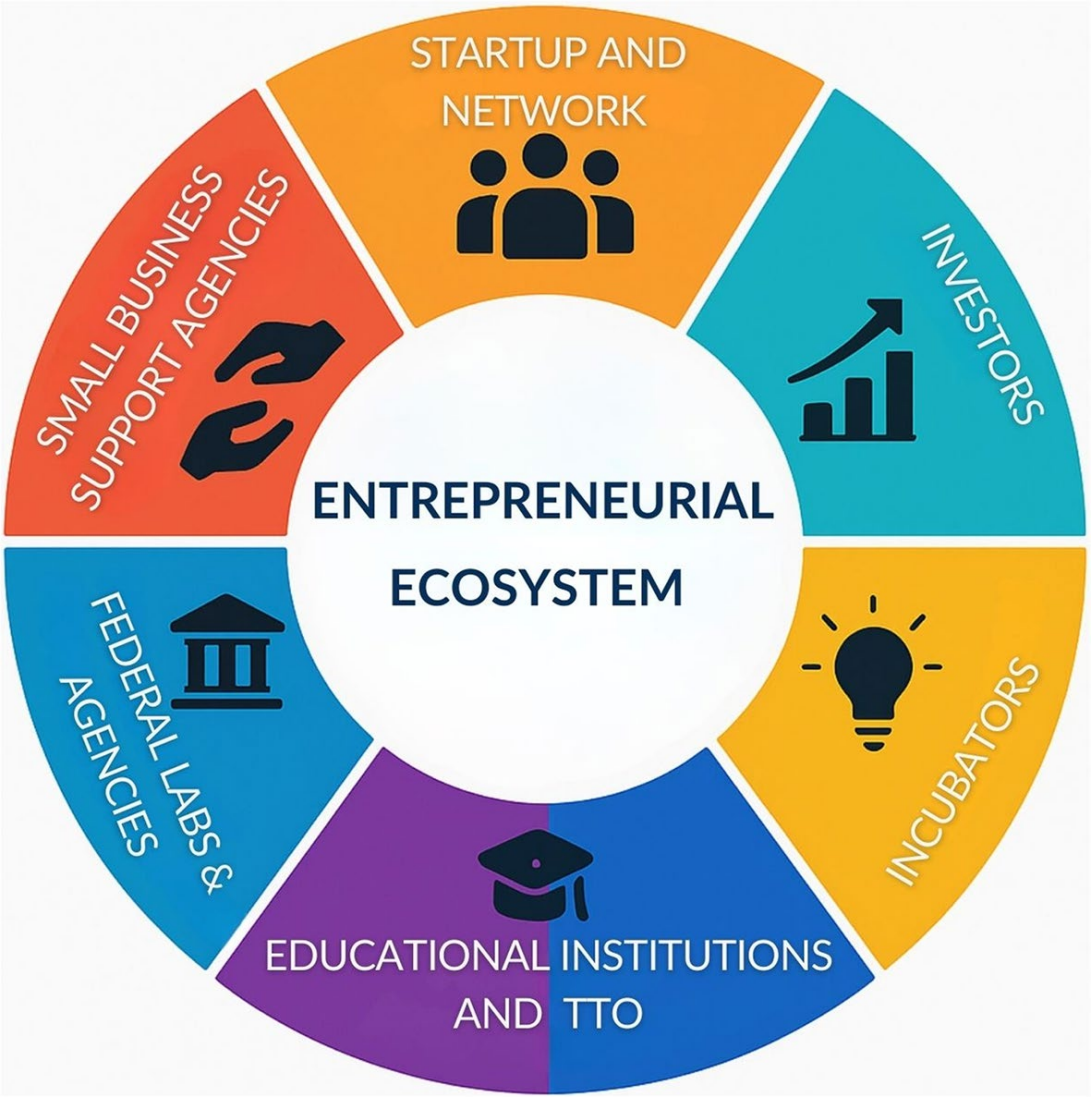
The entrepreneurial ecosystem

The entrepreneurial ecosystem (Fig. 3) consists of the startup, a network of similar startups and entrepreneurs, educational institutions (universities, community colleges, and training centers with their TTOs), federal laboratories and their TTOs, incubators and science parks, major industries, the agencies that help small businesses (i.e., local county or state economic development authorities), chamber of commerce and investor circles. Learning about similar ecosystems across the country may help you decide on the best location for setting up your company. This will be based on identical technology companies in that area, the availability of investors, the skills required in the specific field, and the costs and tax breaks available in the region. It is important to keep in mind that the entrepreneurial ecosystem varies from one area to another and may differ for the products under development, even within the same region: for example, developing a technology solution for patient engagement will have different needs than developing a therapeutic solution for breast cancer. Also, familiarizing oneself with the legal responsibilities (including applicable tax laws) of a company in the state or region where it is formed is essential to maintain the company in good standing.

### Key procedures to establish a small business

When deciding to establish your own business, specific procedures need to be carried out, which may include:

- Company credentials (Name, address, bank account)
- Type of entity (LLC, C-Corp)
- The size of the initial team
- Place of registration to obtain a Tax ID
- Registering in the Federal Grants System [i.e., grants.gov, NIH—eRA Commons, U.S. General Services Administration (SAM.gov)] and the Small Business Administration
- Website (name, domain, email, social media profiles)
- Minority certification, if needed (State and Federal certifications)
- Hiring employees, consultants, and interns for small companies

### Raising different kinds of funds for your company

#### Bootstrapping

Most companies are started by bootstrapping. This means founders use their personal funds and then actively begin to look for funding from additional sources, which may include:

### Small Business Innovation Research (SBIR)/Small Business Technology Transfer Research (STTR) funding

The Small Business Administration (SBA; https://www.sba.gov) administers the SBIR/STTR programs through federal agencies such as the National Institutes of Health (NIH), National Science Foundation (NSF), United States Department of Agriculture (USDA), National Aeronautics and Space Administration (NASA), Department of Energy (DOE), and Department of Defense (DOD). The calls for SBIR/STTR grants are announced on the SBA and each agency’s websites. These grants consist of Phase I – up to $400,000 (depending on the agency) for 6–12 months—and a Phase II grant for up to 2 years and up to $2 M (depending on the agency), with the possibility of securing direct contracts beyond that. These grant announcements come in every year, and the topics of interest are provided in the announcements. Entrepreneurs interested in applying for grants under the SBIR/STTR program should be aware that this program is subject to the federal government appropriations process. Thus, budget changes implemented during that fiscal year may affect the availability of funds or eligibility for these grants. The great advantage of SBIR/STTR funds is that they are a non-dilutive (do not require giving up company equity) source of funding when there is a match in interests. Other opportunities include the availability of matching funds for startups that successfully secure SBIR/STTR funding, thereby increasing the company’s resources for developing the technology [9].

### State grants (i.e., TEDCO, FAST)

Some states offer organization grants to small businesses. An example is the Maryland Technology Development Corporation (TEDCO), which provides funding and connections that early-stage technology and life sciences companies use to thrive in Maryland. Another example is the Federal and State Technology Partnership Program (FAST) in Louisiana (see website below). These are awards to increase the pipeline of SBIR/STTR applicants through training and outreach. Another example of incentives and support is the PyMEs (“Pequeñas y Medianas Empresas”) from the Economic Development and Commerce Department (DDEC) in Puerto Rico. PYMES provides different incentives that may assist companies in their early-stage development. Among these, “PyMEs Innovadoras” offers an incentive to initiate or scale operations with the goal of commercializing and exporting their technology. In many cases, these funds may be small; however, they provide valuable funding and contact with investors. The best way to find local state grant opportunities is to contact your local Small Business Development Center (SBDC), which is part of the U.S. SBA. You can search your local SBDC office by zip code through the SBA website (https://www.sba.gov/local-assistance/resource-partners/small-business-development-centers-sbdc). Furthermore, many SBDC offices offer free training and assistance for your business (e.g., setting up your business portfolio and mentoring opportunities).

### Pitching to Investors and enrolling in competitions

There are investor forums dedicated to specific technology areas to which one can pitch their business idea. A reasonable amount of work toward the product idea should have been accomplished to get here. A few investor forums for the biotech industry are given below:

BIO Investor Forum (https://www.bio.org/calendar/bio-investor-forum) – an international biotech investor conference focused on early and established private companies as well as emerging public companies.

BIO Partnering (https://bigs.bio.org/) – a meeting to navigate the financial landscape for life sciences, tackling regulatory and policy changes, and building relationships with strategic partners for investment and receiving guidance on capital formation, commercialization, reimbursement, and growth.

Biocom (https://www.biocom.org/conferences/global-partnering-conference/) – a partnering and investor forum that brings together over 500 investors and senior executives, including Chief Executive Officers (CEOs) and heads of business development, biotech, and research and development from leading pharmaceutical, biotech, and research organizations from over 20 countries.

California Life Sciences (https://www.califesciences.org/partnering-forum/) – a partnering forum for founders of cutting-edge life science technology startups to connect with investors to advance their products to commercial success.

### Incubators and accelerators

Biotech incubators and accelerators can help you advance your startup to the following stages by providing services, in some cases, for a share (ownership) in the company. “Accelerator” refers to a cohort-based, time-bound growth program, while “incubator” generally provides workspace, long-term support, and commercialization resources. Some universities also support faculty through accelerators and incubators, supporting faculty and students in this venture (see Table A1 in additional files). Usually, these organizations have access to angel investors and venture capital, and as partners, they may bring credibility while pitching for investments.

### Raising private capital (Angel and Seed investors)

Private capital from angel and seed investors usually comes from sources known to the entrepreneur. In return for the investment at a high-risk stage, these investors are typically offered a portion of the company shares. An Angel investor is typically a high-net-worth individual or team that invests personal funds in the early stages of a company. This individual typically offers mentorship and smaller checks. A Seed investor is often an institutional venture capital firm that writes larger checks for a more developed concept. Unique minority angel investor circles and venture funds focused on minority startups may help raise initial capital. The color of money matters! The investors may also offer experience in the field and in business, as well as contacts. Given a choice between an investor who just puts money and an investor who brings all the above additional value, we would choose the latter, an investor with additional value. Mixing events that host investors from all of these organizations is an excellent opportunity for your startup to network.

### Licensing from universities and partnerships

Startups that spin off from inventions created at the university need to license the technology's IP from the University to commercialize the innovation (even if you are named as the inventor of such a patent). The licensing terms should be negotiated between your company and the university, which typically requires legal counsel. Before any commercialization effort in the technology is initiated, these final licensing terms must be provided in writing through a Licensing Agreement. Importantly, in addition to licensing the IP, there must be a clear understanding of the startup's use of university facilities and available resources. If additional licensing and partnering arrangements are needed with other companies, they must also be in writing and duly authorized by the other company's appropriate authority. For Phase II SBIR awards and beyond, funding agencies favorably view partnerships with an established company. Letters of support, contractual agreements, or other formal binding documentation between the entities usually evidence these partnerships.

### Financial knowledge and business models

A good grasp of the basics of startup finance is necessary to make financial estimates and develop business models. Business models have now evolved significantly from the days of pure-selling models. Moreover, business models are dynamic for start-ups – i.e., they can change or pivot over time. The team should be able to consider all scenarios related to the feasibility of generating revenue and profit for the company. The “Lean Business Canvas” is an example of a method for developing or clarifying the company's relations and activities to generate revenue and profit. The lean startup philosophy was created by entrepreneurs Alexander Osterwalder and Yves Pigneur, and is based on a "Business Model Canvas" (BMC). Their approach substitutes the typical 40 + page business plan for a one-page, 9-block canvas that enables entrepreneurs to visually represent, test, and iterate their hypotheses regarding their business, with an ultimate goal of validating their Business Model [10, 11]. This canvas provides a structured way to understand the startup’s business model. There are many models of the lean business canvas, or business model canvas, freely available for download on the internet. Many of the educational programs mentioned earlier include the Lean Business Canvas among their topics.

### Think about your team and your career options

Building a credible team is extremely important for the success of the business. However, before starting the company, check and familiarize yourself with your university’s conflict-of-interest policies. Rules and policies regarding being a university employee and starting or deciding to affiliate yourself with a company (e.g., founder, technical advisor, Chief Technology Officer (CTO), partner, board member, consultant, employee, etc.) must be understood and disclosed. If the university permits any of the above, becoming the founder and CTO might be the best option. Various strengths needed for running the company should be considered when forming the team – leadership and technical skills, business and marketing skills, financial skills, and members with connections to investors. Having an inherently diverse team dramatically enhances the range of thought and perspectives. This diversity may be an added asset while pitching to raise funds. Further, investors invest in a team, not necessarily in the original business idea proposed. The investors and the management team should have an excellent working relationship based on mutual trust. In addition to the technical credibility and soundness of the business idea, the coachability and cohesiveness of the team are critical in raising funds. As a full-time academic, it isn't easy to be a full-time entrepreneur in a start-up, plus it may create conflicts of interest and commitment. Therefore, it is best to hire a full-time CEO and a strong team (research assistants, project manager, accountant, and corporate and patent attorney) to commercialize the innovative technology if the founder decides to maintain their full-time academic position. Some academic founders also take on part-time work at the university to dedicate more time to growing the business. Another option, although not very common, has been done occasionally: to take a sabbatical or a year’s leave of absence, get the company going, and return to academics. However, this may not be sufficient time to fully develop the concept described earlier (Fig. 1).

## Concluding remarks

Biomedical research aims to improve patient outcomes, quality of life, and societal justice. Successful biomedical entrepreneurship bridges laboratory discovery and commercial application in daily life settings – including clinical ones. Therefore, as scientists, we are unequivocally positioned to advance technologies that address unmet needs, particularly those aligned with our scientific and personal experiences. Moreover, we could benefit from successful partnerships among academia, industry, and government agencies to address the most pressing challenges in the biomedical field. Switching to a business environment requires persistence, adaptability, and resilience in a rapidly evolving industry. Seizing the opportunity to transform healthcare through the work developed by *‘our hands’* is one of the most satisfying accomplishments we could achieve in our scientific careers. Therefore, it is increasingly pertinent to provide intellectual property education to the new generation of scientists being formed at universities. Finally, support systems for academic start-ups within and outside universities are also increasingly necessary to facilitate the successful development of viable solutions discovered in the laboratory and to make them accessible to millions of patients and consumers across the globe.

## Short list of favorite books from the authors

1. Academic Entrepreneurship: How to Bring Your Scientific Discovery to a Successful Commercial Product. Author: Michele Marcolongo Ph.D., First published:1 September 2017. Print ISBN:9781118859087 |Online ISBN:9781118859070 |10.1002/9781118859070. © 2017 John Wiley & Sons, Inc.
2. Founders at Work: Stories of Startups' Early Days. Author: Jessica Livingston. Edition: 1st. ISBN-13978–1430210788. Publisher: Apress. Publication date: November 1, 2008.
3. Zero to One: Notes on Startups, or How to Build the Future. Author: Peter Thiel. Edition: 1st. Publisher: Crown Currency. Publication date: September 16, 2014. ISBN-13978–0804139298.
4. From Research to Market: A Comprehensive Guide. Author: Alvaro Ossa. Print ISBN: 9783031713392 | Online ISBN: 9783031713408|. DOI: https://doi.org/10.1007/978–3-031–71340-8 2024. 2024 Springer Cham.
5. Entrepreneurship for Creative Scientists. Authors: Dawood Parker, Surya Raghu and Richard Brooks. Online ISBN: 9780750311465 | Print ISBN: 9780750311472 |DOI: https://doi.org/10.1088/978-0-7503-1146-5. © 2018 IOP Publishing Ltd.
6. Entrepreneurship for Scientists and Engineers. Author: Kathleen Allen. Publication Date: 1st edition (January 18, 2009). Print ISBN: 0132357275. 2009 Pearson.

## Definitions and resources

1. *Commercialization*: entities or individuals interested in using the invention, patent, technology; or the processes related to granting an exclusive or non-exclusive license, equity or any other type of agreement reached with these entities regarding the invention.
2. *Gross Royalties*: royalties derived from the commercialization or invention licenses without any deduction made. These deductions include payments and expenses related to the institution's invention. These expenses include costs incurred in protecting the invention through patents, patent attorney fees, legal fees, and expenses of the process, including licensing the technology or any other cost related to the invention.
3. *Indirect financial investment*: an employee or student’s spouse or dependent child has an economic interest in an entity or benefits by more directly or indirectly of 10% or more of the revenues.
4. *Invention*: any idea, discovery, material, design, process, model, technical development, modification, and transference of these processes or related technology, regardless of them being patentable or eligible for commercialization.
5. *Inventor*: person or persons that create or participate in the creation of a patentable or non-patentable invention.
6. *License*: authorization from the invention’s owner so that a third party can carry out some (non-exclusive) or all (exclusive) rights related to the invention. This includes the right to use, sell, or make an offer to sell the invention.
7. *Licensee*: a person who possesses a license.
8. *Net Royalties*: the remaining royalties after the institution has recovered all of the costs of the invention.
9. *Patent*: a right or title conferred by the United States Patent and Trademark Office, or similar offices in an international setting, that permits the owners, inventors for a set period, the sole right to exclude others from making, using, or selling an invention.
10. *Significant financial interest*: monetary value, including (but not limited to) salary, payment or compensation (payment for consultant services or honorarium), investment interests, or any revenue gained due to intellectual property. This excludes income generated for giving talks, conferences, or educational services sponsored by public or non-profit institutions.
11. *Royalties*: a sum of money paid to a patentee for the use of a patent. It can be awarded upfront or in subsequent installments, as well as in the form of stocks, securities granted by the licensee, or other corporate equity received by the Institution upon licensing the technology or invention. This does not include money from sponsored research agreements or other service contracts.
12. *Non-dilutive funding* refers to financial support provided to a business or organization that does not require the recipient to give up equity or ownership in exchange for the funds. Examples include grants, loans, competitions and awards, tax incentives, and crowdfunding, among others.
13. *TEDCO* (Maryland) https://www.tedcomd.com/about-tedco
14. Federal and State Technology Partnership Program (FAST, Louisiana) https://www.opportunitylouisiana.gov/fast
15. *PyMEs* incentives (Puerto Rico) https://www.desarrollo.pr.gov/ayudas-e-incentivos

## Supplementary Information

Supplementary Material 1.

Supplementary Material 2.

## Data Availability

Not applicable.

## Abbreviations

ACT: Accomplishing Career Transitions
APC: Article Processing Charge
ASCB: American Society for Cell Biology
BMC: Business Model Canvas
CEO: Chief Executive Officer
CTO: Chief Technology Officer
DDEC: Economic Development and Commerce Department
FAST: Federal and State Technology Partnership Program
GMP: Good Manufacturing Practices
IPO: Initial Public Offering
IOP: Institute of Physics
IP: Intellectual Property
IPERT: Innovative Programs to Enhance Research Training
I-RED: IDeA Regional Entrepreneurship Development Program
LLC: Limited Liability Company
MIT: Massachusetts Institute of Technology
NICHD: Eunice Kennedy Shriver National Institute of Child Health and Human Development
NIH: National Institutes of Health
NIGMS: National Institute of General Medical Sciences
NSF: National Science Foundation
PyMEs: Pequeñas y Medianas Empresas
SBA: Small Business Administration
SBDC: Small Business Development Center
SBIR: Small Business Innovation Research
STTR: Small Business Technology Transfer Research
TEDCO: Technology Development Corporation
TTO: Technology Transfer Office
USPTO: United States Patent and Trademark Office

## References

[1] Foster R. From Lab Bench to Startup: How Scientists can Make the Leap. 2025 [cited 2025 December 23]; Available from: https://wewillcure.com/insights/entrepreneurship/translational-research/from-lab-bench-to-startup-how-scientists-can-make-the-leap.

[2] Stokes D. Pasteur’s quadrant: basic science and technological innovation. , e. The Brookings Institution, Editor. 1997, R.R. Donnelley and Sons, Co.: Washington, DC.

[3] Lackeus M. Entrepreneurship in Education: What, Why, When, How in OECD Local Economic and Employment Development (LEED) Papers. Paris: OECD Publishing; 2015.

[4] Jakhar A. What Is Traction In The Startup Context? Here’s All You Need to Know. 2023 [cited 2025 January 25]; Available from: https://inc42.com/glossary/traction/.

[5] Hardymon, F. and T. Nicholas, Kleiner-Perkins and Genentech: When Venture Capital Met Science. 2012. (Revised March 2022.) Harvard Business School Case p. 813–102.

[6] Dayton L. Coronavirus vaccine front-runner Moderna puts MIT chemist-entrepreneur Robert Langer in the spotlight. 2020: Nature.

[7] Patino R. Intellectual property rights and research disclosure in the university environment: preserving the commercialization option and optimizing market interest. J Am Assoc Lab Anim Sci. 2009;48(2):138–43. 19383208 PMC2679667

[8] United States Patent and Trade Office (USPTO). Patents. [cited 2025 December 21]; Available from: https://www.uspto.gov/ip-policy/patent-policy/patents.

[9] Tetlow S. States with SBIR-Matching Funds: Fostering Innovation and Growth. 2024 [cited 12/31/2025; Available from: https://grantengine.com/states-with-sbir-matching-funds-2/.

[10] Osterwalder A. The business model ontology - A proposition in a design science approach.2004, Université de Lausanne.

[11] Osterwalder A, Pigneur Y. Business model generation: A handbook for visionaries, game changers, and challengers. New York: Wiley; 2010.

