# Supplementary material for: Entrepreneurship beyond the lab: commercializing your creative outputs
Source: BMC Proc. 2026 Jun 16;18(Suppl 1):33. doi: 10.1186/s12919-026-00383-3 (PMC13270574; doi:10.1186/s12919-026-00383-3)
Supplement: Supplementary file 1 — Supplementary Material 1. [file 12919_2026_383_MOESM1_ESM.docx]

**Table A1. U.S. university accelerators/incubators that specifically support faculty, researchers, or university-affiliated founders (not exclusively student programs).**

| University | Program Name | Type | Website / More Info |
| --- | --- | --- | --- |
| University of California, Berkeley | SkyDeck *(supports faculty startups alongside students & alumni)* | Accelerator & Incubator | https://skydeck.berkeley.edu/ |
| New York University (NYU) | Tech Venture Program *(faculty/researcher focused entrepreneurship track)* | Accelerator & Support Program | https://entrepreneur.nyu.edu/for-faculty-researchers/ |
| University of New Mexico | Rainforest Accelerator *(faculty & researchers entrepreneurial support)* | Accelerator | https://innovations.unm.edu/program-activities/rainforest-accelerator/ |
| University of Michigan | Innovation Partnerships Startup Incubator *(supports U-M research startups)* | Incubator | https://innovationpartnerships.umich.edu/entrepreneurs/ |
| Purdue University | Purdue Innovates Accelerator *(Purdue-affiliated founders, including faculty)* | Accelerator | https://purdueinnovates.org/incubator/accelerator/ |
| Georgetown University | Georgetown Tech Ventures Incubators & Accelerators *(faculty inventors & tech ventures)* | Incubator & Accelerator | https://otc.georgetown.edu/inventors/supporting-entrepreneurship/georgetown-tech-ventures/ |
| University of Cincinnati | 1819 Venture Lab *(supports faculty & community startup founders)* | Accelerator & Incubator | https://www.innovation.uc.edu/what-we-do/venture-lab.html |
| Harvard University | Harvard Innovation Labs *(open to faculty entrepreneurs and community alongside students)* | Incubator | https://innovationlabs.harvard.edu/ |
| Stanford University | StartX *(open to founders, including faculty/alumni founders via affiliation tracks)* | Accelerator | https://startx.com/ |
| University of North Carolina (UNC) | Innovate Carolina *(venture support & incubator services for university innovators, including faculty)* | Incubator & Venture Support | https://innovate.unc.edu/resources/start-a-venture/ |

Programs may serve multiple founder communities including faculty, alumni, and students, but the above are known to explicitly support faculty or researcher-led ventures through dedicated tracks, eligibility, or open founder policies. Links and programs are active as of February 2026.
