# Supplementary material for: Entrepreneurship beyond the lab: commercializing your creative outputs
Source: BMC Proc. 2026 Jun 16;18(Suppl 1):33. doi: 10.1186/s12919-026-00383-3 (PMC13270574; doi:10.1186/s12919-026-00383-3)
Supplement: Supplementary file 2 — Supplementary Material 2. [file 12919_2026_383_MOESM2_ESM.docx]

**About this supplement**

This article has been published as part of *BMC Proceedings Volume 18 Supplement 1, 2024: Second Accomplishing Career Transitions Workshop 2021.* The full contents of the supplement are available online at <https://bmcproc.biomedcentral.com/articles/supplements/volume-18-supplement-1>.
